# Translational study identifies XPF and MUS81 as predictive biomarkers for oxaliplatin-based peri-operative chemotherapy in patients with esophageal adenocarcinoma

**DOI:** 10.1038/s41598-018-24232-2

**Published:** 2018-05-08

**Authors:** T. P. MacGregor, R. Carter, R. S. Gillies, J. M. Findlay, C. Kartsonaki, F. Castro-Giner, N. Sahgal, L. M. Wang, R. Chetty, N. D. Maynard, J. B. Cazier, F. Buffa, P. J. McHugh, I. Tomlinson, M. R. Middleton, R. A. Sharma

**Affiliations:** 1 NIHR Oxford Biomedical Research Centre, Department of Oncology, University of Oxford, Oxford, UK; 2 Department of Upper GI Surgery, Churchill Hospital, Oxford, UK; 3 Wellcome Trust Centre for Human Genetics, University of Oxford, Oxford, UK; 4 Nuffield Department of Population Health, University of Oxford, Oxford, UK; 5 Medical Research Council Population Health Research Unit (MRC PHRU) at the University of Oxford, Oxford, UK; 6 Ludwig Institute for Cancer Research, University of Oxford, Nuffield Department of Medicine, Oxford, UK; 7 NIHR Oxford Biomedical Research Centre/Department of Cellular Pathology/Radcliffe Department of Medicine, Oxford University Hospitals and University of Oxford, Oxford, UK; 8 Department of Laboratory Medicine, Changi General Hospital, Singapore, Singapore; 9 Laboratory Medicine Programme, University Health Network, Toronto, Canada; 10 Centre for Computational Biology, Institute of Cancer and Genomic Sciences, College of Medical and Dental Sciences, University of Birmingham, Birmingham, UK; 11 Department of Oncology, Weatherall Institute of Molecular Medicine, University of Oxford, Oxford, UK; 12 NIHR University College London Hospitals Biomedical Research Centre, UCL Cancer Institute, University College London, London, UK

## Abstract

Oxaliplatin-based chemotherapy is used to treat patients with esophageal adenocarcinoma (EAC), but no biomarkers are currently available for patient selection. We performed a prospective, clinical trial to identify potential biomarkers associated with clinical outcomes. Tumor tissue was obtained from 38 patients with resectable EAC before and after 2 cycles of oxaliplatin-fluorouracil chemotherapy. Pre-treatment mRNA expression of 280 DNA repair (DNAR) genes was tested for association with histopathological regression at surgery, disease-free survival (DFS) and overall survival (OS). High expression of 13 DNA damage repair genes was associated with DFS less than one year (*P* < 0.05); expression of 11 DNAR genes were associated with worse OS (*P* < 0.05). From clinical associations with outcomes, two genes, *ERCC1* and *EME1*, were identified as candidate biomarkers. In cell lines *in vitro*, we showed the mechanism of action related to repair of oxaliplatin-induced DNA damage by depletion and knockout of protein binding partners of the candidate biomarkers, XPF and MUS81 respectively. In clinical samples from the clinical trial, pre-treatment XPF protein levels were associated with pathological response, and MUS81 protein was associated with 1-year DFS. XPF and MUS81 merit further validation in prospective clinical trials as biomarkers that may predict clinical response of EAC to oxaliplatin-based chemotherapy.

## Introduction

Esophageal cancer is the sixth most common cancer worldwide, and its incidence has increased markedly in recent decades^1–3^. In North America and Europe, adenocarcinoma is the most prevalent subtype^4^.

Cisplatin or oxaliplatin-based combination chemotherapy before surgical resection is currently a standard of care for patients with locally advanced, resectable, HER2-negative esophageal adenocarcinoma (EAC), supported by a meta-analysis^2^ and a Cochrane review. Oxaliplatin-based chemotherapy is as effective as cisplatin-based chemotherapy in advanced esophagogastric cancer, but with lower rates of renal toxicity, neutropenia, alopecia and thromboembolism^5,6^. It is therefore often preferred for patients with co-morbidities, such as the elderly, where it may be given as doublet chemotherapy rather than triplet^7^. In combination with radiation, oxaliplatin-based chemo-radiotherapy is as effective as cisplatin-based chemo-radiotherapy and more convenient for patients^8^.

A concern about offering all resectable patients chemotherapy prior to surgery is our inability to predict which tumors are relatively resistant to treatment and therefore may progress during treatment to become inoperable. We recently showed that major changes can occur in driver mutation presence/frequency in the EAC genome during 2 cycles of oxaliplatin-fluorouracil chemotherapy^9^. Such changes, and epigenetic alterations, can cause rapid alterations to gene expression^10^, which are very likely to affect clinical outcomes. Better selection of patients for the appropriate pre-operative therapy could therefore improve survival rates and quality of life for patients with this disease.

As a translational study between clinical trial and the basic science laboratory, we obtained tumor tissue in a prospective clinical trial of oxaliplatin-fluorouracil chemotherapy in patients with operable EAC. The aim of the hypothesis-generating clinical study was to identify DNA damage repair genes that might predict clinical outcomes. This approach identified 2 candidate biomarkers, which we then studied at a mechanistic level in appropriate cell lines *in vitro*. Building on our previous work showing changes in the EAC genome during chemotherapy, we also compared protein levels of the 2 biomarkers in clinical samples obtained before and after 2 cycles of oxaliplatin-fluorouracil chemotherapy.

## Results

### Clinical outcomes

Thirty-eight patients with confirmed EAC were recruited and had a full dataset available for analysis (Table 1). At a median follow-up of 21.3 months, 28 patients (57.1%) had died. Kaplan-Meier estimates of median OS and DFS were 23.8 months (95% CI 14.8 to 33.0 months) and 17.8 months (95% CI 7.4 to 27.6 months) respectively, consistent with published studies^11,12^.

**Table 1:** Clinical characteristics of patients in the study.

Characteristic	Oxaliplatin-fluorouracil cohort (N = 38)		Surgery-alone cohort (N = 54)	
**Age**				
Median	67		64	
Range	49–78		40–80	
**Sex**				
Male	30	(73.3%)	46	(85.2%)
Female	8	(26.7%)	8	(14.8%)
**Clinical stage**				
I	9	(23.7%)	17	(31.5%)
IIa	6	(15.8%)	7	(13.0%)
IIb	6	(15.8%)	5	(9.3%)
III	17	(44.7%)	25	(46.3%)
IV	0	(0%)	0	(0%)

Table shows the comparison of age, sex, and clinical stage for the oxaliplatin-fluorouracil treated patients in the clinical trial, and a separate cohort of patients treated with surgery alone.

### DNA repair gene mRNA expression in clinical samples before and after chemotherapy

High pre-treatment expression of 7 DNAR genes demonstrated a potential association with lack of pathological response (*P* < 0.05) and 13 DNAR genes were potentially associated with DFS less than one year (*P* < 0.05) following oxaliplatin-fluorouracil chemotherapy (Table 2). These included two genes known to play an important role in repairing inter-strand cross-links (ICLs) induced by platinum chemotherapies, *ERCC1* (log fold-change (FC) −0.624, *P* = 0.025) and *EME1* (logFC −0.573, *P* = 0.037). Higher levels of expression of 11 DNAR genes were associated with worse OS (Table 2) (P < 0.05), with the most significant relationship per unit increase being for *ERCC1* (Hazard Ratio [HR] 3.21; 95% CI [1.55, 6.22]; *P* = 0.001). None of these results reached significance when adjusted for multiple testing; the data were used for hypothesis testing to identify potential candidate genes using a raw *P*-value of less than 0.05. No significant associations were found between DNAR genes and pathological T-stage, N-stage, age or sex.

**Table 2:** Significant associations between pre-treatment DNAR gene expression and pathological response, disease free survival or overall survival.

Gene	LogFC		*P*-value
**Higher expression associated with lack of pathological response**			
*MPG*	−4.035		0.010
*MEN1*	−3.024		0.010
*USP3*	−2.880		0.020
*ALKBH1*	−3.288		0.025
*MGMT*	−1.383		0.028
*PARP3*	−2.543		0.035
*KIN*	−2.154		0.041
**Higher expression associated with DFS <1 year**			
*CHAF1B*	−0.648		0.004
*BCCIP*	−0.704		0.005
*RAD51L1*	−0.597		0.014
*ESCO1*	−0.639		0.015
*RDM1*	−0.689		0.020
*MEN1*	−0.599		0.022
*ERCC1*	−0.624		0.025
*H2AFX*	−0.503		0.027
*NEIL3*	−0.581		0.027
*EME1*	−0.573		0.037
*ALKBH1*	−0.421		0.041
*RAD51*	−0.561		0.046
*GTF2H4*	−0.359		0.046
**Gene**	**Hazard ratio (With 95% CI)**		***P*** **-value**
**Higher expression associated with worse OS**			
*ERCC1*	3.113	(1.56–6.22)	0.001
*ERCC6*	10.505	(1.71–64.55)	0.011
*HINFP*	13.008	(1.52–111.2)	0.019
*RAD51L1*	3.611	(1.23–10.58)	0.019
*HMGB1*	4.381	(1.26–15.26)	0.020
*ALKBH1*	5.174	(1.28–20.98)	0.021
*FSBP*	3.907	(1.18–8.11)	0.021
*NEIL1*	4.161	(1.19–14.52	0.025
*GTF2H2*	3.865	(1.16–12.84)	0.027
*ESCO2*	2.796	(1.05–7.42)	0.039
*BCCIP*	2.634	(1.01–6.90)	0.049

Principal component analysis was carried out to ensure that tumor and non-malignant epithelium showed segregation and to check for any technical artifacts. Differential expression (log fold change) of DNAR genes in tumor tissue from 38 patients with esophageal adenocarcinoma was compared between pathological non-responders and pathological responders, and between patients with DFS <1 year and DFS >1 year, using linear regression. Pre-treatment gene expression levels significantly associated with OS were identified by Cox proportional hazards regression. Unadjusted P values are presented to enable identification of all hits appropriate for hypothesis testing and subsequent validation, albeit with recognized risk that some of the hits might appear by chance.

**Figure 1:**
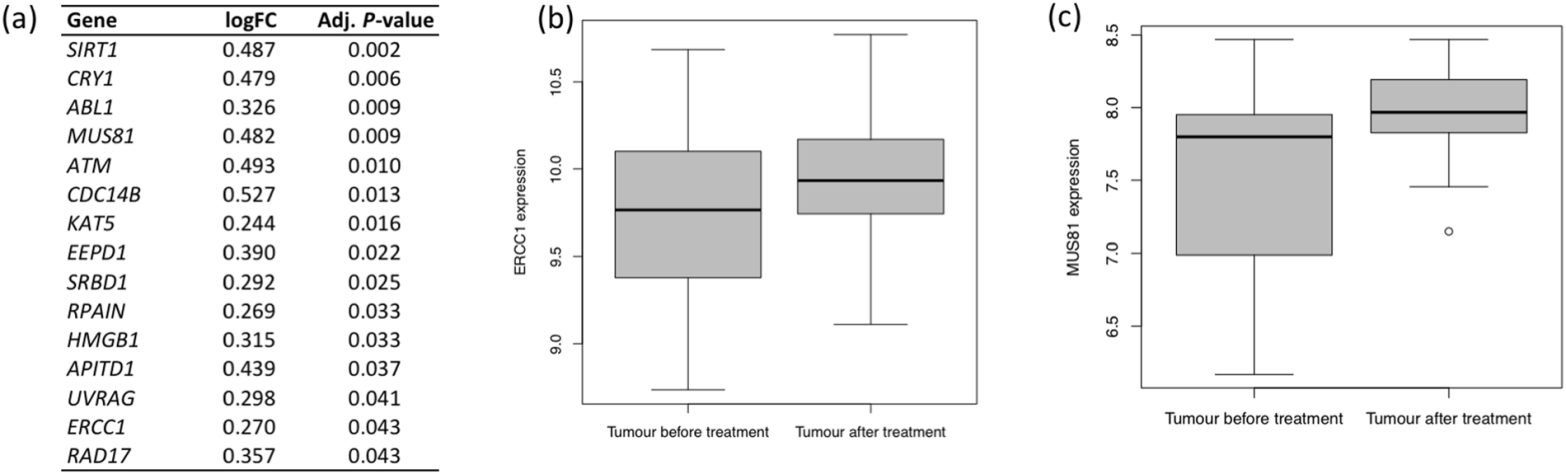
Expression of DNA repair genes in EAC tissue following two cycles of oxaliplatin-fluorouracil chemotherapy. (**a**) Significantly increased expression (*P* values adjusted for multiple testing <0.05) for log fold-change (FC) of 15 genes including *ERCC1* and *MUS81*. (**b**,**c**) Significant changes shown by box and whisker plot for: (**b**)*ERCC1* mRNA expression (logFC 0.270, adjusted *P* = 0.043); (**c**)*MUS81* mRNA expression (log FC = 0.482, adjusted *P* = 0.009).

Based on our recent demonstration of major changes in the EAC genome during 2 cycles of oxaliplatin-fluorouracil chemotherapy^9^, we studied expression of DNAR genes in post-chemotherapy biopsy specimens compared with pre-chemotherapy specimens. We found that expression of 15 DNAR genes was significantly increased following oxaliplatin-fluorouracil (adjusted for multiple testing, *P*-value < 0.05) (Fig. 1a). These included two genes whose pre-chemotherapy expression levels were associated with worse clinical outcomes: *ERCC1* (logFC 0.270, adjusted *P* = 0.043) (Fig. 1b) and *MUS81* (log FC = 0.482, adjusted *P* = 0.009) (Fig. 1c), the binding partner for *EME1* (see below).

### ERCC1 mRNA expression correlates with XPF protein levels in clinical samples

Based collectively on the significant findings outlined above and genes known to play a part in ICL repair, two substrate-specific heterodimeric endonucleases, XPF/ERCC1 and MUS81/EME1, were identified as candidate biomarkers. These proteins have structure-specific activity at junctions between single-strand and double-strand DNA^13^. Subunits of both endonucleases exhibit interdependent stability, with expression levels of each subunit regulated by its heterodimeric partner^14,15^. For IHC purposes, the available EME1 and ERCC1 antibodies lack specificity, and do not differentiate active ERCC1 isoforms^16^. To validate the mRNA expression data for *ERCC1* and *EME1* at the protein level, we instead used antibodies to XPF and MUS81, having first demonstrated high specificity and validity of these antibodies by Western blot and IHC in *XPF* and *MUS81* siRNA treated cells (Supplementary Figures S1 and S2). Correlation of pre-treatment tumor mRNA expression with XPF and MUS81 protein expression, measured by IHC composite score, showed a statistically significant association between *ERCC1* mRNA expression and XPF protein levels (*P* = 0.041) in the clinical samples.

### Association of XPF and MUS81 protein levels with clinical endpoints

**Figure 2:**
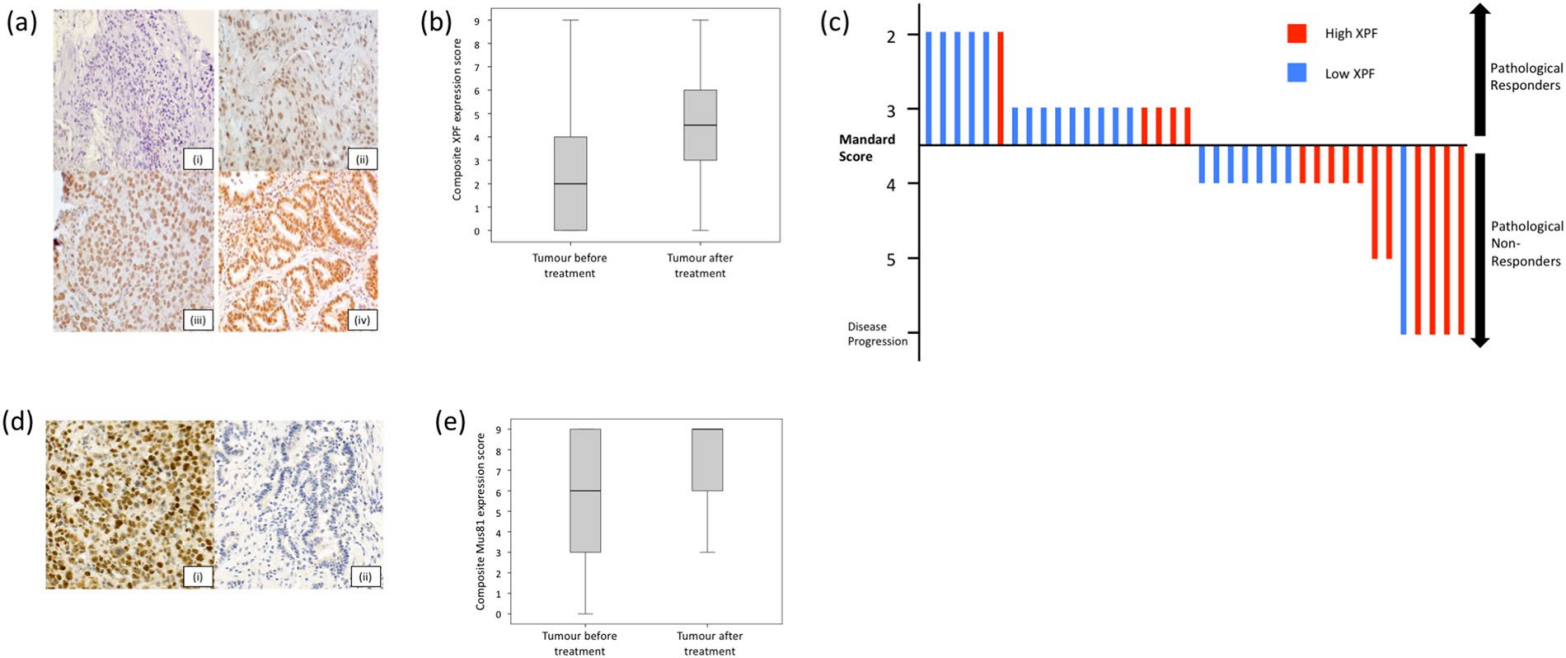
XPF and MUS81 protein levels in EAC tissue by immunohistochemistry. (**a**) Esophageal adenocarcinoma pre-treatment biopsies stained for XPF protein, photographed at x20 magnification; intensity scores (i) 0, (ii) 1, (iii) 2 and (iv) 3. (**b**) Significant increase in XPF protein levels when pre-treatment biopsies were compared with post-treatment resection samples following 2 cycles of oxaliplatin-fluorouracil chemotherapy (median pre-treatment 2, IQR 0–4; median post-treatment 4.5, IQR 3–6; *P* = 0.004, Wilcoxon signed rank test). (**c**) A waterfall plot of the association between pre-treatment tumour XPF level and pathological response following oxaliplatin-chemotherapy. Each line represents the degree of Mandard regression in an individual patient. Patients exhibiting Mandard grades 1–3 were classified as pathological responders; those with Mandard grades 4 and 5 were classified as non-responders^10^. Low XPF expression was associated with pathological response (odds ratio 3.85, *P* = 0.041). (**d**) MUS81 staining of esophageal adenocarcinoma pre-treatment biopsies, photographed at X20 magnification, showing examples of (i) high and (ii) low expression. (**e**) Trend towards increased MUS81 staining when pre-treatment biopsies were compared with post-treatment resection samples following 2 cycles of oxaliplatin-fluorouracil chemotherapy (median pre-treatment 6, IQR 3–9; median post-treatment 9, IQR 6–9; *P* = 0.051, Wilcoxon signed rank test).

Adjusting for prognostic variables, age, stage and gender, we studied the protein levels of XPF and MUS81 in pre-treatment tumor biopsies to derive potential relationships with clinical outcomes. Typical examples of pre-treatment biopsy staining are shown in Fig. 2a for XPF, and Fig. 2d for MUS81. Low XPF protein levels were associated with pathological response to oxaliplatin-fluorouracil chemotherapy (Fig. 2c) (OR = 3.85, *P* = 0.049). High MUS81 protein levels were associated with worse 1-year DFS (OR = 5.00, *P* = 0.04) and worse OS (median 15.5 months *vs*. not reached, *P* = 0.013).

We also compared XPF and MUS81 protein levels before and after 2 cycles of chemotherapy. As demonstrated for ERCC1 mRNA expression, there was a significant increase in XPF staining (Fig. 2b) (median pre-treatment 2, IQR 0–4; median post-treatment 4.5, IQR 3–6; *P* = 0.004, Wilcoxon signed rank test). For MUS81, there was a non-significant increase in staining in the post-treatment samples (Fig. 2e).

### XPF and MUS81 protein levels in a retrospective surgical cohort

To check whether our findings from the clinical trial cohort related to prediction of response to oxaliplatin-fluorouracil chemotherapy, rather than a prognostic effect, we looked for an association between XPF or MUS81 protein levels and OS in a cohort of patients who received surgery alone for EAC, frequency matched by clinical characteristics to the oxaliplatin-fluorouracil cohort (Table 1). Adjusting for prognostic variables, age, stage and gender, no association was observed between XPF or MUS81 protein levels and OS in the surgery-alone cohort of patients. These data suggest that neither of the proteins studied is a prognostic biomarker for patients with EAC who do not receive platinum-based chemotherapy prior to surgery.

### XPF, ERCC1 and MUS81 are not commonly mutated in EAC

Since the mutational statuses of *XPF*, *ERCC1* and *MUS81* in EAC have not previously been reported in detail, we performed Whole Exome Sequencing (WES), in samples obtained pre-chemotherapy from 30 patients. No pathogenic somatic mutations were detected. In one patient, there was an increase in somatic copy number of *ERCC1* (ratio 1.82). Based on this subset analysis, we speculate that *XPF* and *MUS81* are not commonly mutated in EAC. IHC may therefore represent a suitable means of detecting functional protein levels for this disease.

### Cells deficient in XPF or MUS81 protein levels are more sensitive to oxaliplatin treatment

**Figure 3:**
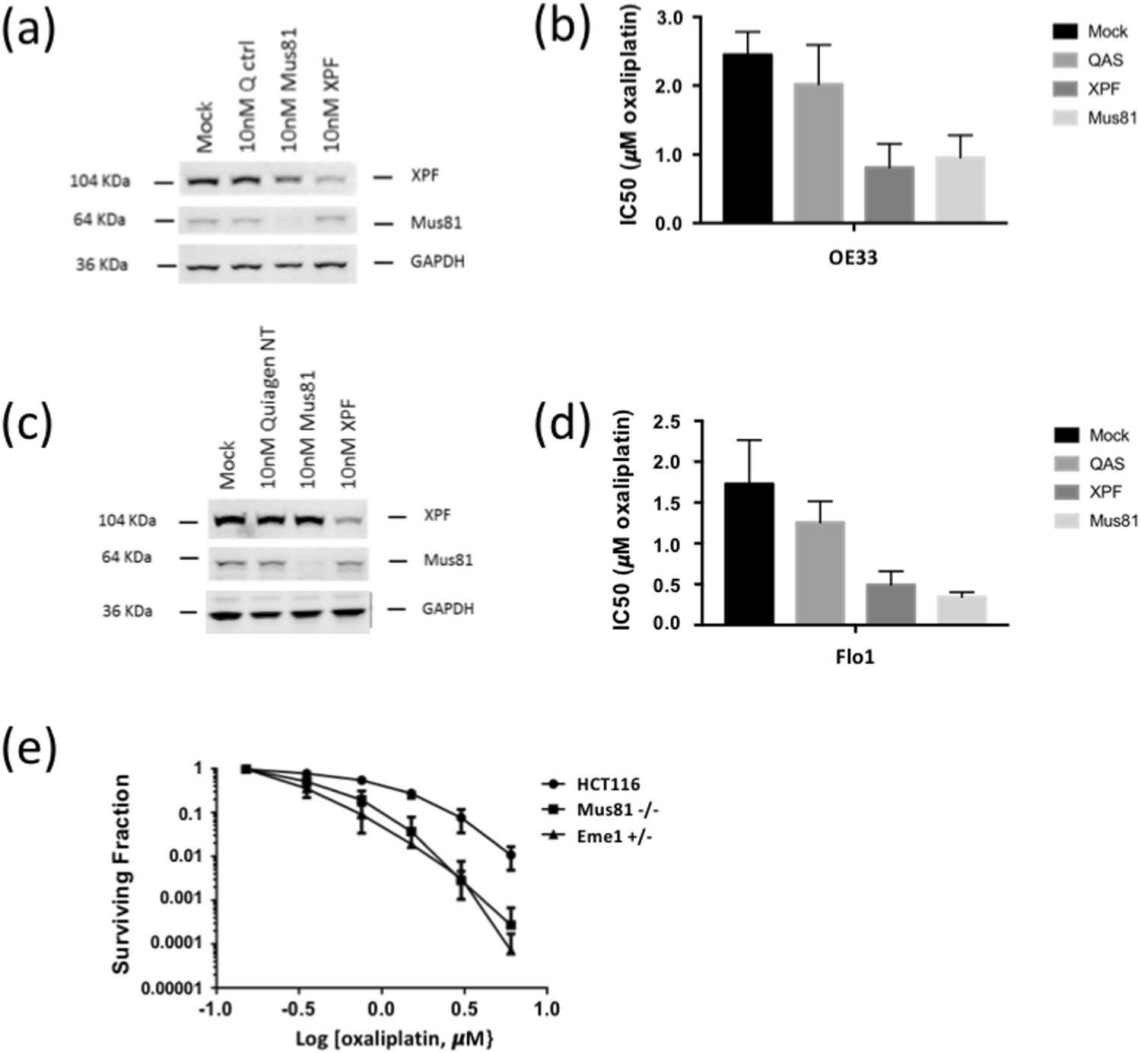
Oxaliplatin sensitivity is increased when cancer cells are deficient in MUS81, XPF or EME1. (**a**–**f**) siRNA depletion of MUS81 or XPF in two EAC cell lines (**a**,**b**) OE33 cells and (**c**,**d**) Flo1 cells. In OE33 cells, oxaliplatin IC_50_ was 2.46 μM (±0.35) in mock-transfected, 2.02 μM (±0.47) in control-transfected, 0.92 (±0.35, *P* < 0.01) after MUS81 depletion and 0.77 (±0.33, *P* < 0.01) after XPF depletion. For Flo1, oxaliplatin IC_50_ was 1.70 μM (±0.47) in the mock- and 1.23 μM (±0.23) in the control-transfected cells, 0.2 (±0.05, *P* < 0.01) after MUS81 depletion and 0.34 (±0.18, *P* < 0.01) after XPF depletion. (**e**) Clonogenic survival of HCT116 colorectal cancer cells is significantly reduced in *MUS81* knockout or *EME1* haplo-insufficient variants.

Finally, to obtain mechanistic *in vitro* corroboration of the observations from the clinical data, we measured the oxaliplatin sensitivity of cell lines deficient in XPF, MUS81 and EME1. Oxaliplatin IC_50_ measured by cell proliferation assay was up to 3-fold higher in the mock and control transfected cells (*P* < 0.01), compared with either the XPF-depleted or the MUS81-depleted cell lines (Fig. 3a–d). In genetically altered *MUS81* and *EME1* variants of the HCT116 cell line, oxaliplatin sensitivity of the parental cell line was more than twofold higher than the MUS81 knockout and EME1 haploinsufficient variants (*P* < 0.05), confirmed by clonogenic survival assays (Fig. 3e).

## Discussion

The development and validation of predictive biomarkers to guide rational selection of pre-operative therapy for individual patients should be a high priority to reduce the frequency with which patients are treated with inappropriate chemotherapy and to improve survival rates for patients with EAC.

In this translational study, tumor mRNA levels of 280 DNA damage repair genes were measured before and after two cycles of oxaliplatin-fluorouracil chemotherapy for 38 patients with EAC. The clinical study was hypothesis-generating, with the aim being to identify DNA repair genes for development as predictive biomarkers for the objective selection of patients for platinum chemotherapy. Unlike retrospective cohort studies limited by the amount of diagnostic material available, particular strengths of this study included the availability of sufficient tissue to obtain a full complement of mRNA scores before and after treatment, WES data for a subgroup of the cohort and validation of two mechanistically justified biomarkers at the protein level.

When association of DNA repair gene expression with clinical endpoints was investigated, high levels of *ERCC1* mRNA expression were associated with worse one-year DFS and OS. In order to validate these mRNA expression findings with semi-quantitative protein measurement in matched tissue samples, we attempted to study ERCC1 protein levels by IHC. As previously highlighted, the commercially available anti-ERCC1 antibodies were insufficiently specific and failed to differentiate between isoforms of ERCC1, leading to unacceptable risk of false positive data^16^. Since XPF/ERCC1 and MUS81/EME1 are heterodimeric endonucleases, with each subunit tightly regulating levels of its binding partner^14,15^, we demonstrated the specificity of the anti-XPF and anti-MUS81 IHC as surrogates for anti-ERCC1 and anti-EME1. Using this method, we demonstrated a significant relationship between *ERCC1* mRNA expression levels and XPF protein levels in our clinical cohort. This is an important finding, justifying the use of XPF IHC as a means of measuring expression of the XPF-ERCC1 heterodimer in clinical samples. High levels of XPF protein were associated with lack of pathological response, and high levels of MUS81 protein were associated with worse DFS and OS.

No association was detected between expression of XPF and prognosis in a matched cohort of patients treated with surgery alone, supporting the potential development of this biomarker as predictive of response to platinum-based chemotherapy. This finding is consistent with a meta-analysis in non-small cell lung cancer patients, which showed that *ERCC1* expression is not a prognostic biomarker for patients treated with surgery alone^17^.

Our results are consistent with previous findings on treatment failure following oxaliplatin-based chemotherapy of EAC. In an important study, Leichman *et al*. demonstrated an inverse relationship between intra-tumoral *ERCC1* mRNA expression and progression-free and overall survival in patients with operable esophageal cancer receiving neoadjuvant oxaliplatin-fluorouracil and radiation therapy^18^. Our findings suggest that high levels of *ERCC1* mRNA are associated with poor DFS and OS in a similar subject group.

MUS81 is an appealing target for cancer therapy on account of its extensive network of synthetic lethalities in preclinical model systems^19^. Our data are consistent with the known mechanism of action of MUS81 and our corroboration of the mechanism *in vitro* (Fig. 3). It is currently thought that XPF/ERCC1 is responsible for making the initial incisions that initiate ICL repair, while MUS81/EME1 is utilised to process an intermediate that persists when the XPF-dependent pathway fails^20^, hence the value of using two levels of the same pathway as mechanistic biomarkers. The inter-dependence between ICL repair pathway proteins is of interest in target discovery to sensitise cancers to platinum therapy as well as in biomarker development for patient selection in which panels of mechanistically selected biomarkers are likely to be superior to single biomarkers used in isolation^21^.

Polymorphisms of ERCC1 and XPF have been reported in Chinese patients with esophageal cancer, potentially influencing clinical outcomes following platinum-based chemotherapy^22,23^. We analysed exome sequencing data from 30 patients in our cohort. The somatic sequences observed do not suggest that mutations in *XPF*, *MUS81* and *ERCC1* are common in EAC, leading us to conclude that immunodetection of XPF and MUS81 by IHC is a reasonable strategy for detecting protein levels that may impact on repair of platinum-induced DNA damage. In a related study, we showed major changes in mutation presence or frequency could occur in EAC following 2 cycles of pre-operative chemotherapy, and we proposed that these ‘clonal shifts’ could be arise from selection of drug-resistant cancer cells during treatment^9^. We extended this analysis to the induction of DNA damage repair genes following chemotherapy, and found higher expression of 15 genes, including *ERCC1* and *MUS81*, and higher XPF and MUS81 protein staining. Since we have shown that MUS81 deficiency and XPF deficiency are both associated with sensitivity to oxaliplatin chemotherapy, it is reasonable to speculate that cells within a tumor that have high expression of MUS81 or XPF will have a greater chance to clonally evolve and to survive through treatment. This might explain the significant induction of *ERCC1* mRNA and XPF protein following chemotherapy, and similar trends with MUS81. These data indicate the need to monitor the tumor during therapy; it is hoped that developments in circulating tumor biomarkers will allow such monitoring via a blood test in the future.

In conclusion, our translational study has identified 2 DNA damage repair proteins that are potential biomarkers for predicting clinical response to oxaliplatin-fluorouracil chemotherapy. To support this hypothesis, we have combined gene expression data analysis with mechanistic assays of oxaliplatin-induced DNA damage, and we have used IHC to further evaluate the candidate biomarkers identified. We advocate validation of XPF and MUS81 in a large, independent cohort of EAC patients, to enable their development as potential biomarkers to identify patients who will benefit from oxaliplatin-based chemotherapy.

## Methods

### Patients

The clinical study was approved by the Oxfordshire Regional Ethics Committee and all methods were performed in accordance with the relevant guidelines and regulations, with registrations: prospectively with EudraCT 2005-000834-34 and retrospectively with ISRCTN18146225 (date of assignment 12.10.2017). All participants had World Health Organisation (WHO) performance status 0–2 and provided written informed consent. Recruitment occurred between May 2006 and February 2010. Individuals over the age of 18 with histologically proven invasive cancer of the esophagus or esophagogastric junction (EGJ), as per Union for International Cancer Control (UICC) definition of esophageal cancer (6^th^ edition), but excluding Siewert type III EGJ tumors, were eligible for inclusion. Exclusion criteria included: stage I (T1 N0) disease or any evidence of metastatic disease. See Supplementary Information.

A matched cohort of 54 EAC patients treated with surgery alone between 1997 and 2004 was used to study if either biomarker identified in the study was prognostic in the absence of peri-operative chemotherapy. Patients were frequency matched for age, sex and stage, as shown in Table 1.

### Investigations and Treatment

Patients had clinical examination, full blood count and measurement of serum biochemistry at baseline and before each cycle of chemotherapy. Pre-treatment staging was performed by contrast-enhanced computed tomography (CT), ^18^fluorodeoxyglucose (FDG-) positron emission tomography (PET)-CT, and endoscopic ultrasound. Staging laparoscopy was performed for all patients with tumors involving the esophago-gastric junction, and distal esophageal tumors extending below the diaphragm. Pre-treatment tumor and normal tissue samples (from macroscopically normal squamous epithelium 5–10 cm proximal to the upper limit of visible tumor) were biopsied endoscopically, and samples obtained from surgery in those proceeding to surgical resection. Samples were placed immediately into RNAlater solution, for later RNA extraction, or formalin, for subsequent processing into paraffin blocks, or flash frozen in liquid nitrogen. A strict sampling handling protocol was mandatory, with uniform fixation times, tissue processing and storage conditions for all samples in the study.

Patients received two cycles of oxaliplatin-fluorouracil chemotherapy. A cycle of treatment lasted 21 days and consisted of oxaliplatin (130 mg/m^2^ IV) on day 1, followed by fluorouracil (1000 mg/m^2^/day IV) on days 1–4. Restaging was performed by ^15^F-FDG PET-CT. Patients who did not progress to become inoperable were operated on 4–6 weeks after completion of chemotherapy. Pathological response was determined using the histological grading of tumor regression described by Mandard^24^. All slides were independently reviewed by two specialist gastrointestinal pathologists, with 96% concordance^25^.

### RNA quantification

Gene expression was measured in the tumor biopsies and post-surgical resection samples. RNA was extracted using the Qiagen AllPrep DNA/RNA Micro Kit (Qiagen Inc., Valencia, CA) following the manufacturer’s instructions. RNA purity was verified via NanoDrop spectrophotometer (Thermo Fisher Scientific, Waltham, MA). Extracted mRNA samples were hybridised to Illumina HumanHT-12-v3 Expression BeadChips (Illumina, San Diego, CA). Raw gene expression data was processed using Illumina GenomeStudio software (v1.6) to generate expression intensities which were considered in a logged base 2 scale. Data were then analysed using R statistical software (v2.15) (http://www.Rproject.org) (R development Core Team, 2012) with BioConductor packages (Gentleman *et al*., 2004), limma (v3.12.3)(Smyth, 2005), vsn (v3.24) and survival (v2.36.13), for microarray analyses and SPSS software version 19.0 (IBM Inc., Armonk, NY, USA) for all other analyses. A P-value of <0.05 was considered statistically significant. Differential expression between the experimental groups was assessed by generating relevant contrasts corresponding to the two-group comparison. Hazard ratios for OS and DFS according to gene expression level were calculated using Cox regression (see Statistical methods section below). For functional pathway analysis, the Gene Set Analysis (GSA) R package was used to determine the significance of pre-defined sets of genes (the biocarta gene sets) with respect to outcome variables. Finally, a hypothesis-driven approach was undertaken and validation focused on the differentially expressed genes amongst 280 genes belonging to DNA damage repair pathways expected to be relevant for the treatment response (Supplementary Table S1).

### Immunohistochemistry

Immunohistochemistry (IHC) was carried out using the Leica Bond-Max automated immunostainer (Leica Microsystems, Wetzlar, Germany), using the manufacturer’s protocol. IHC was conducted on 5 μm sections cut from paraffin-embedded biopsies, stained with mouse anti-XPF (clone SPM228) at 1:200 dilution or mouse anti-MUS81 (clone MTA30 2G10/3) at 1:1000 dilution, both from Abcam, Cambridge, MA). Antibodies to XPF, MUS81 and ERCC1 (Santa Cruz sc-17809) were tested for specificity using cell pellets depleted for XPF or MUS81 protein by siRNA interference (Supplementary Figures S1 and S2). For XPF measurement, two independent assessors scored staining semi-quantitatively via a score relating nuclear staining intensity and the percentage of stained nuclei. For MUS81, high expression was defined by a median cutoff, greater than 10% of nuclei staining with high intensity. Further details available in Supplementary Information.

### Whole exome sequencing

Thirty EAC patients were prioritized for whole exome sequencing (WES) on the basis of pathological response, disease free survival (DFS) and overall survival (OS) at 2 years, performed as previously described^9^.

### Cell lines and *in vitro* experiments

Colorectal cancer cell line HCT116 and isogenic variants (*MUS81* null and *EME1* haplo-insufficient)^25^ were provided by Prof. Kiyoshi Miyagawa (University of Tokyo, Japan). Esophageal cell lines OE33 and Flo1 were obtained from ECACC. For siRNA depletion, cells were treated 96 and 48 hours prior to plating using ON-TARGETplus siRNA (GE Lifesciences, Dharmacon, USA) to *MUS81* and *XPF*, and Lipofectamine® RNAiMAX transfection reagent (Thermofisher), following manufacturers’ instructions (full details in Supplementary Information).

### Statistical methods

For definitions of disease-free survival (DFS) and overall survival (OS), see Supplementary Information. Individuals were censored at the last date they were known to be alive and disease-free (DFS), or alive (OS). Kaplan-Meier curves were calculated for DFS and OS. Due to the relatively small number of individuals in the study and in order not to exclude potentially useful candidate genes, analyses of the relationship between pre-treatment gene expression levels and clinical outcomes were not corrected for multiple testing. Individual gene expression was tested for association with pathological response, one year DFS and OS, and a *P*-value of <0.05 was considered statistically significant. For comparison of pre- and post- treatment gene expression, *P*-values were adjusted for multiple testing using the Benjamini and Hochberg (1995) procedure^26^.

## Novelty and impact

We performed a translational clinical trial to discover biomarkers for esophageal adenocarcinoma (EAC) patients who are likely to benefit from oxaliplatin-based chemotherapy. Through mRNA expression profiling, we identified DNA repair genes associated with clinical outcomes. Two novel biomarkers were identified and studied by immunohistochemistry of clinical samples and mechanistic studies in cancer cell lines. These biomarkers merit further validation in prospective clinical trials in patients with EAC.

## Electronic supplementary material

Supplementary Information
