# Supplementary material for: Translational study identifies XPF and MUS81 as predictive biomarkers for oxaliplatin-based peri-operative chemotherapy in patients with esophageal adenocarcinoma
Source: Sci Rep. 2018 May 8;8:7265. doi: 10.1038/s41598-018-24232-2 (PMC5940885; doi:10.1038/s41598-018-24232-2)
Supplement: Supplementary file 1 — Supplementary Information [file 41598_2018_24232_MOESM1_ESM.pdf]

Translational study identifies XPF and MUS81 as predictive biomarkers for oxaliplatin-based peri-operative chemotherapy in patients with esophageal adenocarcinoma

T. P. MacGregor[1], R. Carter[1], R. S. Gillies[1,2], J. M. Findlay[2,3], C. Kartsonaki[1,4,5], F. Castro-Giner[3], N. Sahgal[6], L. M. Wang[7,8], R Chetty[9], N. D. Maynard[2], J. B. Cazier[1,10], F. Buffa[1], P. J. McHugh[11], I. Tomlinson[3], M. R. Middleton[1]*, R. A. Sharma[1,12]*

1 NIHR Oxford Biomedical Research Centre, Department of Oncology, University of Oxford, Oxford, UK

2 Department of Upper GI Surgery, Churchill Hospital, Oxford, UK

3 Wellcome Trust Centre for Human Genetics, University of Oxford, UK

4 Nuffield Department of Population Health, University of Oxford, Oxford, UK

5 Medical Research Council Population Health Research Unit (MRC PHRU) at the University of Oxford, Oxford, UK

6 Ludwig Institute for Cancer Research, University of Oxford, Nuffield Department of Medicine, Oxford, UK

7 NIHR Oxford Biomedical Research Centre/Department of Cellular Pathology/Radcliffe Department of Medicine, Oxford University Hospitals and University of Oxford, UK

8 Department of Laboratory Medicine, Changi General Hospital, Singapore

9 Laboratory Medicine Programme, University Health Network, Toronto, Canada

10 Centre for Computational Biology, Institute of Cancer and Genomic Sciences, College of Medical and Dental Sciences, University of Birmingham, UK

11 Department of Oncology, Weatherall Institute of Molecular Medicine, University of Oxford, UK

12 NIHR University College London Hospitals Biomedical Research Centre, UCL Cancer Institute, University College London, UK

* Equal contribution

Corresponding author: Professor Ricky Sharma, NIHR University College London Hospitals Biomedical Research Centre, UCL Cancer Institute, University College London, UK

Email: ricky.sharma@oncology.ox.ac.uk

Supplementary Figures and Tables

Supplementary Table S1

Gene	Entrez I.D.	Gene	Entrez I.D.	Gene	Entrez I.D.	Gene	Entrez I.D.	Gene	Entrez I.D.	Gene	Entrez I.D.	Gene	Entrez I.D.	Gene	Entrez I.D.
ABL1	25	CETN2	1069	EYA1	2138	INTS3	65123	NSMCE2	286053	PRMT6	55170	RPA1	6117	TOP2A	7153
ALKBH1	8846	CHAF1A	10036	EYA2	2139	JMY	133746	NTHL1	4913	PRPF19	27339	RPA2	6118	TOPBP1	11073
ALKBH2	121642	CHAF1B	8208	EYA3	2140	KAT5	10524	NUDT1	4521	PTTG1	9232	RPA3	6119	TP53	7157
ALKBH3	221120	CHD1L	9557	EYA4	2070	KIN	22944	OBFC2A	64859	RAD1	5810	RPA4	29935	TP53BP1	7158
APEX1	328	CHEK1	1111	FANCA	2175	LIG1	3978	OBFC2B	79035	RAD17	5884	RPAIN	84268	TREX2	11219
APEX2	27301	CIB1	10519	FANCB	2187	LIG3	3980	OGG1	4968	RAD18	56852	RPS27L	51065	TRIP13	9319
APITD1	378708	CINP	51550	FANCD2	2177	LIG4	3981	PARG	8505	RAD21	5885	RPS3	6188	TTC5	91875
APTX	54840	CLSPN	63967	FANCE	2178	MBD4	8930	PARP1	142	RAD23A	5886	RRM2B	50484	TYMS	7298
ASF1A	25842	CRY1	1407	FANCF	2188	MC1R	4157	PARP2	10038	RAD23B	5887	RTEL1	51750	UBE2A	7319
ASTE1	28990	CRY2	1408	FANCG	2189	MDC1	9656	PARP3	10039	RAD50	10111	RUVBL2	10856	UBE2B	7320
ATM	472	CSNK1D	1453	FANCI	55215	MEN1	4221	PARP4	143	RAD51	5888	SETMAR	6419	UBE2D3	7323
ATR	545	CSNK1E	1454	FANCL	55120	MGMT	4255	PCNA	5111	RAD51AP1	10635	SETX	23064	UBE2N	7334
ATRIP	84126	CUL4A	8451	FANCM	57697	MLH1	4292	PIPSL	266971	RAD51C	5889	SFPQ	6421	UBE2V1	7335
ATRX	546	CUL4B	8450	FBXO18	84893	MLH3	27030	PMS1	5378	RAD51L1	5890	SHPRH	257218	UBE2V2	7336
ATXN3	4287	DCLRE1A	9937	FBXO6	26270	MNAT1	4331	PMS2	5395	RAD51L3	5892	SIRT1	23411	UHRF1	29128
AXIN2	8313	DCLRE1B	64858	FEN1	2237	MORF4L1	10933	PMS2CL	441194	RAD54B	25788	SLC30A9	10463	UIMC1	51720
BARD1	580	DCLRE1C	64421	FSBP	10646	MORF4L2	9643	PMS2L1	5379	RAD54L	8438	SLK	9748	UNG	7374
BAZ1B	9031	DDB1	1642	FTO	79068	MPG	4350	PMS2L11	441263	RAD9A	5883	SLX1B	79008	UPF1	5976
BCCIP	56647	DDB2	1643	FZR1	51343	MRE11A	4361	PMS2L2	5380	RBBP8	5932	SMC1A	8243	USP1	7398
BLM	641	DNA2	1763	GADD45A	1647	MSH2	4436	PMS2L5	5383	RBM14	10432	SMC3	9126	USP10	9100
BRCA1	672	DTL	51514	GADD45G	10912	MSH3	4437	PNKP	11284	RBX1	9978	SMC6	79677	USP3	9960
BRCA2	675	EEPD1	80820	GEN1	348654	MSH5	4439	POLA1	5422	RDM1	201299	SMG1	23049	UVRAG	7405
BRCC3	79184	EME1	146956	GIYD1	548593	MSH6	2956	POLB	5423	RECQL	5965	SMUG1	23583	VCP	7415
BRE	9577	EME2	197342	GTF2H1	2965	MTMR15	22909	POLD1	5424	RECQL4	9401	SOD1	6647	WDR33	55339
BRIP1	83990	EPC2	26122	GTF2H2	2966	MUM1	84939	POLD3	10714	RECQL5	9400	SRBD1	55133	WRN	7486
BTBD12	84464	ERCC1	2067	GTF2H3	2967	MUS81	80198	POLD4	57804	REV1	51455	SSRP1	6749	WRNIP1	56897
BTG2	7832	ERCC2	2068	GTF2H4	2968	MUTYH	4595	POLE	5426	REV3L	5980	STRA13	201254	XAB2	56949
C1orf124	83932	ERCC3	2071	GTF2H5	404672	NBN	4683	POLE2	5427	RFC1	5981	SUMO1	7341	XPA	7507
CCNH	902	ERCC4	2072	H2AFX	3014	NCOA6	23054	POLG	5428	RFC2	5982	SUPT16H	11198	XPC	7508
CCNO	10309	ERCC5	2073	HELQ	113510	NEIL1	79661	POLG2	11232	RFC3	5983	SUPT6H	6830	XRCC1	7515
CDC14B	8555	ERCC6	2074	HERC2	8924	NEIL2	252969	POLH	5429	RFC4	5984	TDG	6996	XRCC2	7516
CDK7	1022	ERCC8	1161	HINFP	25988	NEIL3	55247	POLK	51426	RFC5	5985	TDP1	55775	XRCC3	7517
CDKN2D	1032	ESCO1	114799	HMGB1	3146	NHEJ1	79840	POLL	27343	RFWD3	55159	TERF2IP	54386	XRCC5	7520
CEBPG	1054	ESCO2	157570	HMGB2	3148	NONO	4841	POLQ	10721	RNF168	165918	TMEM161A	54929	XRCC6	2547
CEP164	22897	EXO1	9156	IGHMBP2	3508	NSMCE1	197370	PRKDC	5591	RNF8	9025	TNP1	7141	XRCC6BP1	91419

Table S1. List of DNA repair genes studied in microarray analysis
Note that XPF protein is encoded by the ERCC4 gene.

Supplementary Figure S1

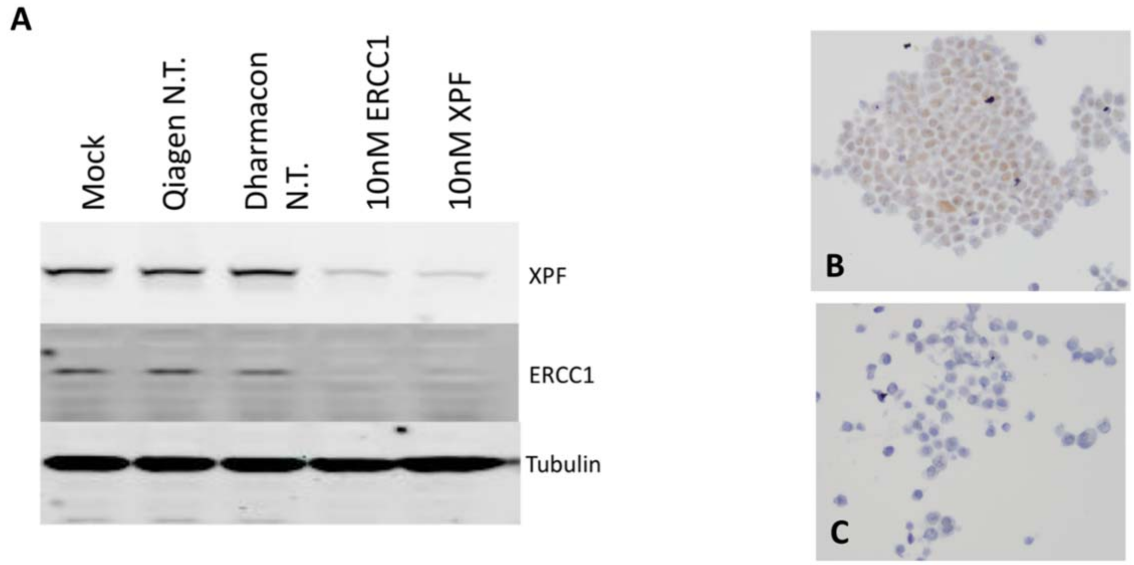

Supplementary Figure S1. Specificity of SPM228 antibody for detection of XPF protein.
(A) Western blot showing specificity of SPM228 antibody against XPF and mutual stabilization of XPF and ERCC1 in ST16 (gastric cancer cell line) cells. siRNA knockdown of XPF markedly decreased ERCC1 detection. Likewise, ERCC1 knockdown reduced XPF protein levels. (B) Measurement of XPF as a surrogate for ERCC1 levels by IHC, with high levels detected in mock transfected cells. (C) Decreased IHC staining for XPF in ST16 cells following siRNA knockdown of XPF.

Supplementary Figure S2

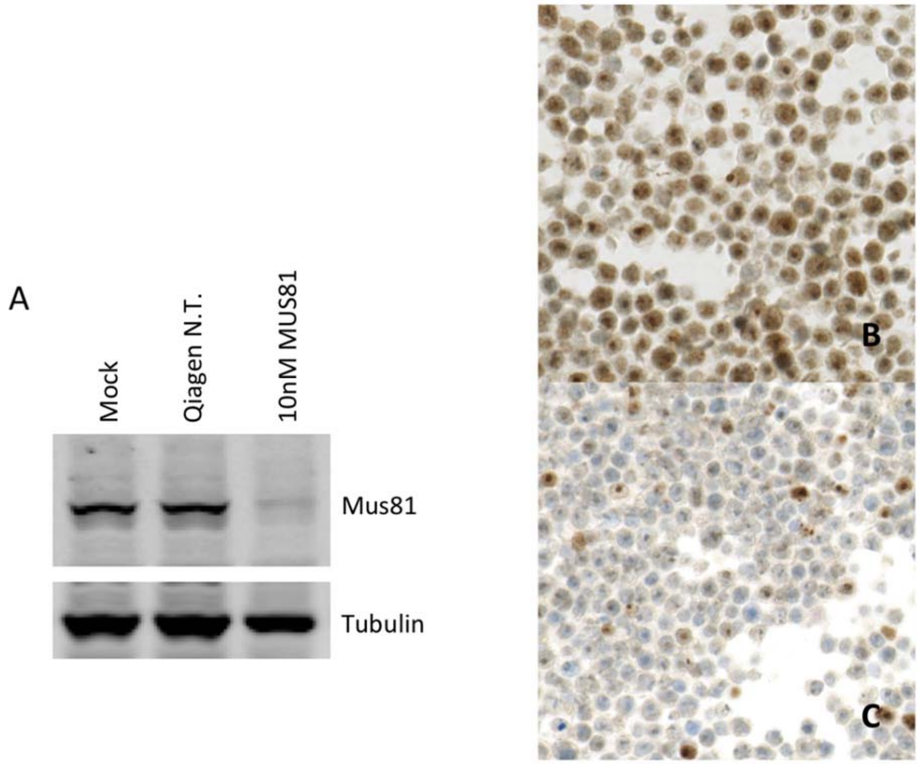

Supplementary Figure S2. Specificity of MTA30 2G10/3 antibody for detection of MUS81 protein.

Specificity of MTA30 2G10/3 antibody against MUS81 demonstrated by (A) Western blot showing significant decrease in MUS81 following siRNA knock-down of MUS81 in ST16 cells. (B&C) The same cells fixed for IHC staining, showing: (B) strong staining in mock transfected cells, compared to (C) decreased staining for MUS81 following siRNA knockdown of MUS81.

Supplementary Figure S3

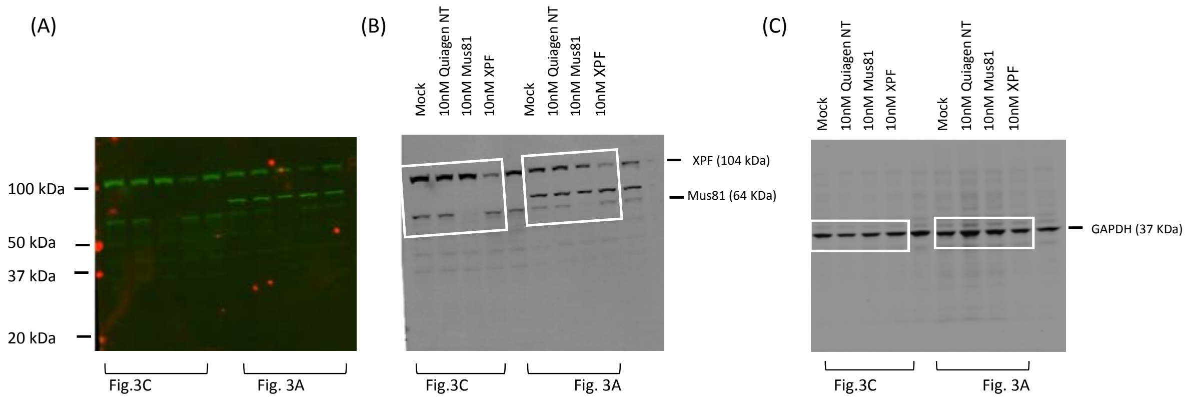

Supplementary Figure S3. Full picture of Western blot cropped to form Figure 3 (A and C). (A) Full picture of blot, showing marker, probed with mouse primary antibodies to Mus81 and XPF, detected with Alexa Fluor® 680 conjugated goat mouse antibody. (B) The same blot shown greyscale, as in Figure 3A and C. (C) The same blot with rabbit primary antibody to GAPDH, detected with Alexa Fluor® 790 conjugated goat anti-rabbit antibody.

Supplementary Figure S4

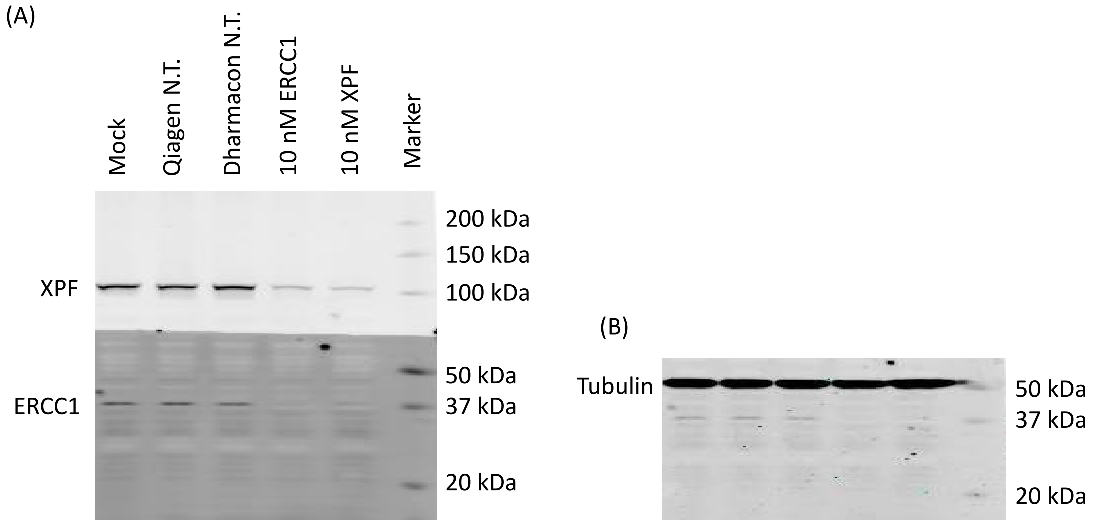

Supplementary Figure S4. Full picture of Western blot cropped to form supplementary figure S1. (A) The upper section of the blot was probed with mouse primary antibodies to XPF, detected with Alexa Fluor® 680 conjugated goat mouse antibody. The lower section of the blot was probed with mouse anti-ERCC1, detected with Alexa Fluor® 680 anti-mouse antibody. (B) the lower section of the blot probed with rabbit primary antibody to tubulin, detected with Alexa Fluor® 790 anti-rabbit antibody.

Supplementary Figure S5

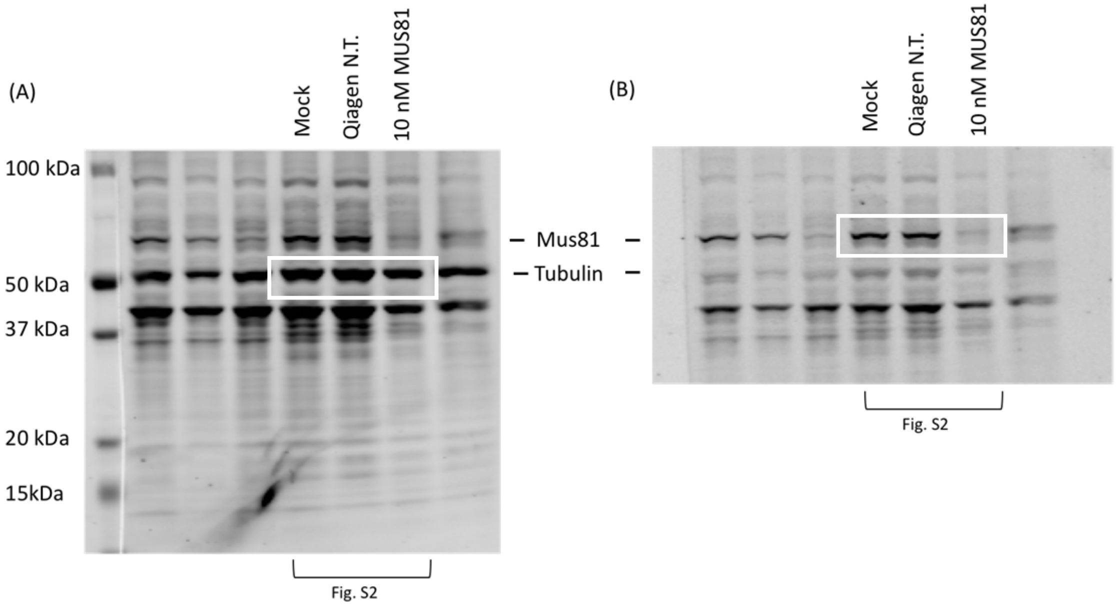

Supplementary Figure S5. Full pictures of Western blot cropped to form supplementary figure S2A. The blot was probed with mouse primary antibodies to Mus81 and tubulin, detected with Alexa Fluor® 680 conjugated goat anti-mouse antibody.

(A) High exposure (with marker) used to show tubulin, (B) low exposure, used to show Mus81.
